# Case Report: Advanced colorectal cancer diagnosed and treated with chemotherapy during pregnancy

**DOI:** 10.3389/fonc.2025.1699233

**Published:** 2025-12-02

**Authors:** Arianna Galante, Marco Cerbone, Giuseppe Sorgente, Pasquale Moramarco, Tommaso Difonzo, Gennaro Cormio, Marco Marinaccio, Raffaello Alfonso, Ettore Cicinelli, Antonella Vimercati

**Affiliations:** 1 Unit of Obstetrics and Gynecology, Department of Interdisciplinary Medicine (DIM), University of “Aldo Moro”, Polyclinic of Bari, Bari, Italy; 2 Unit of Oncologic Gynecology, IRCCS “Giovanni Paolo II” Oncologic Institute, Bari, Italy

**Keywords:** cancer, pregnancy, pregnancy associated cancer, colorectal cancer, chemotherapy, chemotherapy in pregnancy

## Abstract

**Introduction:**

Cancer during pregnancy is a rare event and often presents at an advanced stage due to delayed diagnosis. Clinical symptoms are frequently misattributed to normal pregnancy changes, leading to diagnostic challenges. Moreover, concerns regarding fetal safety limit the use of certain imaging modalities and treatment options. Managing cancer in pregnancy requires careful coordination across specialties to balance maternal treatment with fetal preservation.

**Case-report:**

We present the case of a pregnant woman diagnosed in the second trimester with advanced metastatic colorectal cancer. The disease involved multiple intra-abdominal sites, and the patient was managed through a multidisciplinary approach. Chemotherapy with FOLFOX scheme was administered during the second and third trimester of pregnancy, leading to a favorable clinical and radiologic response. Delivery was planned at term, with no complications for the newborn. Postpartum oncologic management was continued without delay.

**Conclusion:**

This case highlights the importance of individualized care in such complex scenarios and the feasibility and safety of administering chemotherapy during pregnancy.

## Introduction

Pregnancy-associated cancer (PAC), defined as cancer diagnosed during pregnancy or within one year postpartum, presents a rare but complex clinical scenario with significant implications for both maternal and fetal health. (1) The coexistence of pregnancy and malignancy introduces unique challenges in diagnosis, management, and ethical decision-making. (2) Due to the rarity of PAC and the exclusion of pregnant patients from most clinical trials, evidence-based treatment guidelines are limited, and therapeutic strategies are often based on expert opinion, case reports, and retrospective studies. (3, 4).

It is estimated that approximately 1 in every 1,000 to 2,000 pregnancies is complicated by a cancer diagnosis. (5) The most commonly encountered malignancies during pregnancy are breast cancer, melanoma, and cervical cancer, which collectively account for the majority of PAC cases. In contrast, colorectal cancer (CRC) in pregnancy is exceedingly rare, constituting only 1–3% of all pregnancy-related cancers. (6).

Diagnosing CRC during pregnancy can be particularly challenging, as symptoms such as nausea, vomiting, constipation, and abdominal discomfort may be misattributed to normal gestational changes, often resulting in delayed diagnosis and more advanced disease at presentation. (7, 8) Furthermore, management options must be carefully balanced to optimize maternal outcomes while minimizing fetal risks. (9, 10) Chemotherapy is contraindicated during the first trimester due to teratogenicity but may be considered in the second and third trimesters. (11, 12) Surgical interventions, when indicated, are generally safe throughout pregnancy, whereas radiotherapy is typically deferred until after delivery due to substantial fetal risk. (13).

With the increasing trend of early-onset CRC and the rise in maternal age, the incidence of CRC diagnosed during pregnancy is expected to grow. (14) In this context, we report a rare and instructive case of a pregnant woman diagnosed with CRC, highlighting the diagnostic challenges, multidisciplinary decision-making, and therapeutic approach tailored to both maternal and fetal considerations.

## Case report

A 37-year-old Albanian pregnant patient, with no prior medical or surgical history and a negative family history for oncological disease, was referred for an abdominal MRI due to persistent abdominal pain unresponsive to medication for over a month. Symptoms were initially self-managed with over-the-counter analgesics, as she believed they were pregnancy-related. However, due to worsening discomfort and increasing abdominal girth, she sought medical evaluation. At that time, she was 21 weeks pregnant with her second child (the first pregnancy uneventful). She reported that routine prenatal examinations performed in Albania were described to her as normal, although only limited documentation was available for review.

While residing in her home country, the patient underwent an abdominal MRI, which revealed an enlarged liver (right lobe measuring approximately 23 cm), with multiple focal lesions in both hepatic lobes, suggestive of metastases, ranging in size from 1 to approximately 10 cm. There were also multiple peritoneal and omental thickenings. Ascites filled all peritoneal recesses. No lymphadenopathy was identified. Bilateral adnexal masses were also observed and initially interpreted as possible primary ovarian malignancies.

The patient elected to continue diagnostic evaluation and treatment in Italy and was referred to our unit at approximately 24 weeks of gestation. An abdominal ultrasound confirmed diffuse ascites, peritoneal thickenings forming an omental cake-like appearance, a solid pelvic mass on the left side of uncertain origin, possible intestinal or ovarian relevance, measuring up to 12 cm in maximal diameter, poorly vascularized, and another pelvic lesion on the right side, likely originating from the right adnexa. Multiple hepatic lesions were also noted, consistent with metastatic disease. Gynecologic evaluation raised suspicion of a gastrointestinal primary tumor with widespread metastases. The original MRI from Albania was reviewed by a local radiologist, who confirmed the suspicion of a bowel-origin neoplasm.

Upon admission to our center in Italy, the patient presented in poor general conditions, with severe anemia (hemoglobin level was 7.5 g/dL), dispnea, and abdominal pain due to large volume of ascites. She underwent evacuative paracentesis and received two blood transfusions, along with corticosteroids for fetal lung maturation. Electrocardiogram showed sinus rhythm. Laboratory investigations showed elevated tumor markers (AFP 95.4 ng/mL, CEA 2183 ng/mL, CA15.3–55 U/mL, CA125–418 U/mL, and CA19.9–400 U/mL), while platelet count, white cell count, electrolytes, liver and renal function tests, and coagulation profile were within normal limits. Obstetric ultrasound revealed no signs of fetal distress and the estimated fetal weight (EFW) at first assessment was at the 30th percentile (Hadlock charts). A liver biopsy confirmed the diagnosis of gastrointestinal malignancy.

During hospitalization, the patient underwent rectosigmoidoscopy, which revealed an infiltrative lesion at the rectosigmoid junction. Histological examination of biopsy specimens confirmed the diagnosis of metastatic adenocarcinoma of intestinal origin, moderately differentiated (G2), with immunohistochemistry positive for CDX2 and CK20, and negative for CK7 and PAX8.

The case was discussed at a multidisciplinary tumor board involving obstetricians, neonatologists, oncologists, and psychologists. It was decided to initiate chemotherapy during pregnancy, starting at 26 weeks of gestation. The patient received a total of five biweekly cycles of FOLFOX (oxaliplatin 85 mg/m² + 5-FU bolus 400 mg/m²) from week 26 to week 34. The patient tolerated chemotherapy well, with no treatment-related adverse events such as neuropathy, thrombocytopenia, or gastrointestinal toxicity. Supportive therapy consisted of ondansetron for nausea, and no additional supportive measures were required. Serial ultrasound assessments demonstrated preserved fetal Doppler studies and biophysical parameters, although a progressive growth restriction emerged, with the estimated fetal weight decreasing to the 5th percentile at 37 weeks.

**Figure 1:**
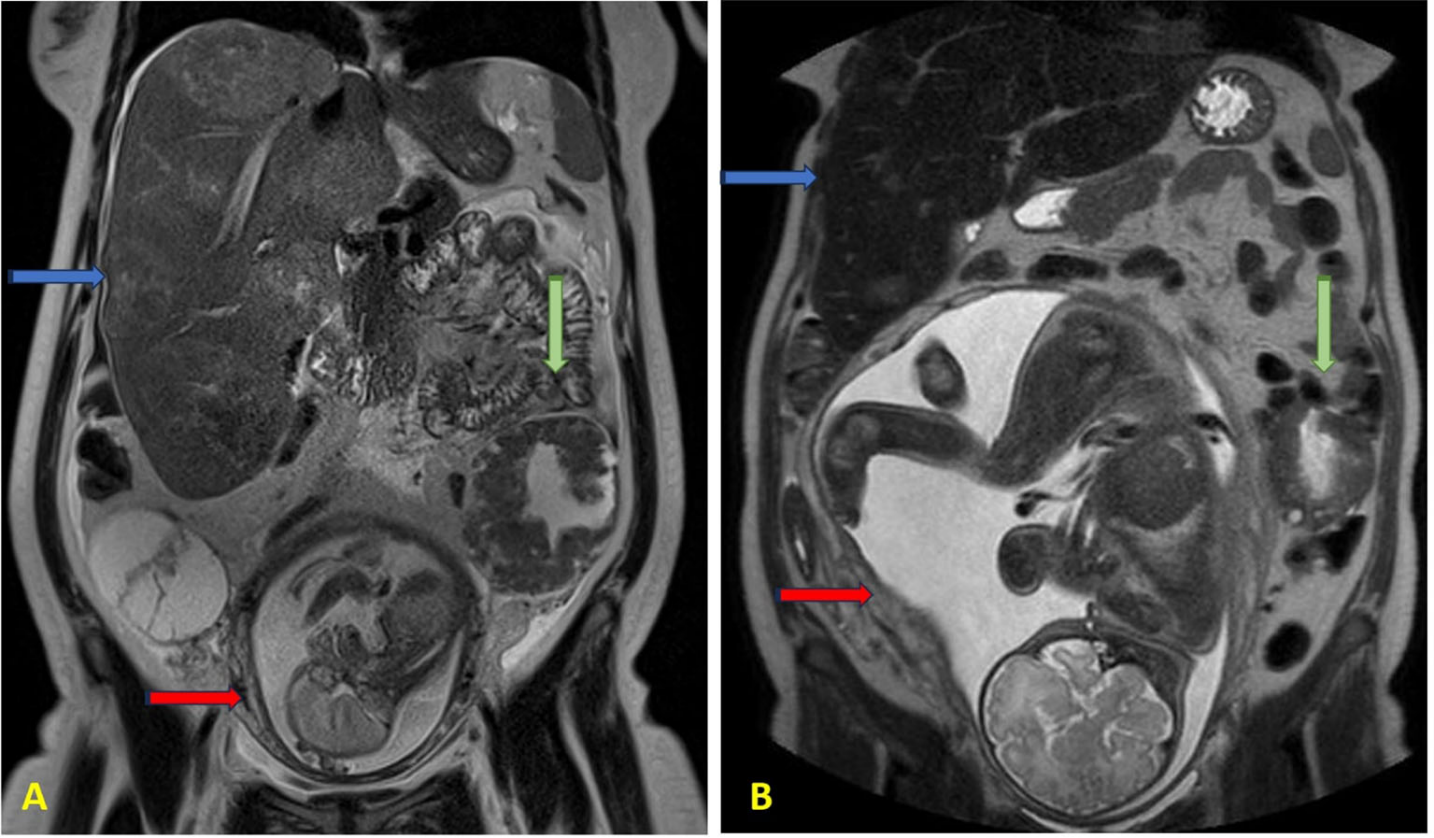
Abdomen MRI at **(A)** 21 weeks of gestation and **(B)** 37 weeks of gestation. The blue arrows indicate enlarged liver containing multiple metastatic lesions; the green arrows indicate the left pelvic mass; the red arrows indicate the fetus in the uterus.

**Figure 2:**
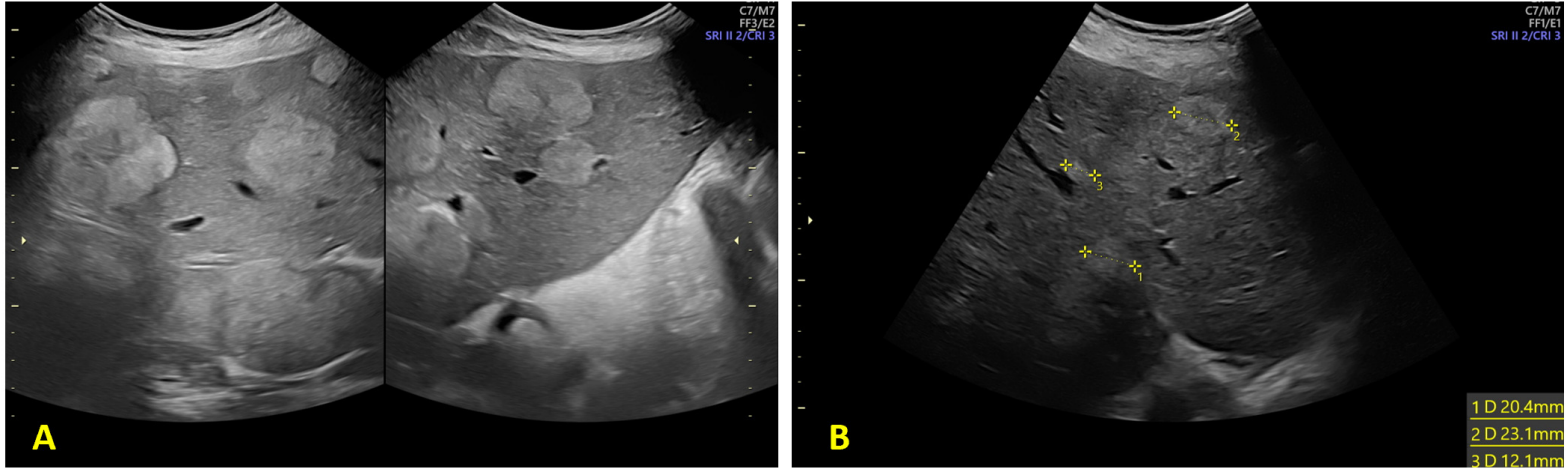
Ultrasound evaluation of hepatic metastasis **(A)** 28 weeks of gestation and **(B)** at 33 weeks of gestation.

Following completion of five chemotherapy cycles, the tumor marker levels showed marked reduction: AFP 78 ng/mL, CEA 189 ng/mL, CA15.3–46 U/mL, CA125–20 U/mL, and CA19.9–59 U/mL. Radiological re-evaluation thruough MRI documented a significant reduction in the number and size of hepatic metastases. Peritoneal carcinomatosis was no longer visible and ascites had resolved (**Figure 1**) On ultrasound evaluation the largest hepatic lesion decreased from 10 cm to 6 cm, and all previously identified liver nodules showed a reduction in size of at least 50%. (**Figure 2**) The pelvic masses had also substantially decreased in size. (**Table 1**).

**Table 1:** Table summarizing tumor markers and imaging findings at initial evaluation (25 weeks) vs post-chemotherapy (34 weeks).

Parameter	At initial evaluation (25 weeks)	After chemotherapy (34 weeks)	Change/Observation
AFP (ng/mL)	95.4	78	↓
CEA (ng/mL)	2183	189	↓
CA19.9 (U/mL)	400	59	↓
CA125 (U/mL)	418	20	↓
CA15.3 (U/mL)	55	46	↓
Liver findings (MRI/US)	Multiple metastases in both hepatic lobes (1–6 cm, largest 10 cm); hepatomegaly (23 cm right lobe)	Reduction in number and size of hepatic lesions (largest 6 cm); >50% overall reduction in size	↓Partial radiological response
Peritoneal findings (MRI/US)	Multiple peritoneal and omental thickenings (“omental cake”); diffuse ascites	No visible peritoneal carcinomatosis; resolution of ascites	↓ Regression
Pelvic mass (MRI/US)	Left-sided solid mass, right adnexal lesion; poorly vascularized	Marked reduction in size of lesions	↓ Significant reduction
Overall assessment	Advanced metastatic disease	Partial response to treatment with significant regression of lesions	—

A second multidisciplinary discussion was held to determine the timing and mode of delivery. It was agreed to deliver the fetus via elective caesarean section (CS) at 37 weeks of gestation - ensuring a three-week interval from the last chemotherapy cycle - with concurrent bilateral adnexectomy.

The CS was performed at 37 weeks and 4 days, delivering a female infant weighing 2355 g. Neonatal condition was good: arterial pH was 7.28, base excess (B) -1.2 mmol/L, extracellular base excess (BEecf) 0.1 mmol/L, and Apgar scores were 9 and 10 at 1 and 5 minutes, respectively. The newborn showed no signs of distress during hospitalization. After thorough counseling with the neonatology team regarding the risks and benefits of breastfeeding in her oncologic context, the patient opted to suppress lactation.

**Figure 3:**
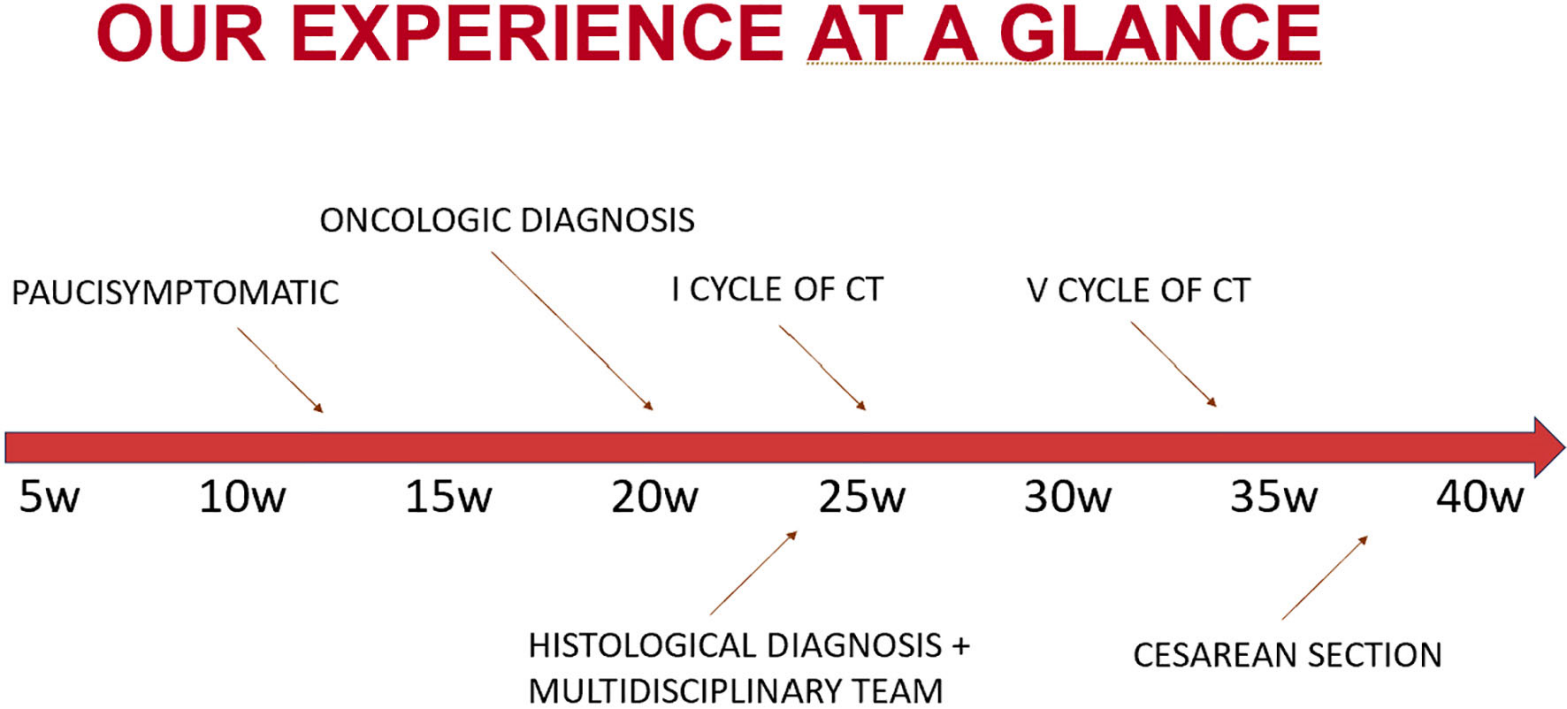
A brief timeline of our experience.

Bilateral adnexectomy was performed during the procedure. Histopathology confirmed metastatic adenocarcinoma involvement in both ovaries (CDX2+, CK20+, CK7–, PAX8–, WT1–; HER2 score 0; microsatellite stability detected). Examination of the placenta, membranes, and umbilical cord revealed no tumor infiltration. Both fallopian tubes were free of malignancy. The postoperative course was uneventful, the patient underwent radiologic reassessment and continued first-line chemotherapy as per oncologic plan (**Figure 3**).

## Discussion

Colorectal cancer during pregnancy remains a rare and challenging diagnosis, particularly due to symptom overlap with physiological changes of pregnancy, and concerns over fetal exposure during imaging and treatment. In our case, early symptoms were underestimated by the patient causing a delay in medical evaluation. Once referred to our unit, however, diagnostic work-up and treatment were initiated without delay, as our center has established experience in managing cancer during pregnancy.

In the differential diagnosis of a pelvic mass on ultrasound, it is important to consider the possibility of a pelvic abscess or infectious complication in pregnant women - particularly in presence of systemic symptoms such as fever, pelvic pain, asthenia, and vomiting. (15, 16) Pelvic abscesses typically present with more irregular margins, internal content consisting of dense fluid collections with debris, thin septations or fluid-fluid levels, and tend to show low and peripheral vascularization on Doppler imaging. Additionally, compression with the ultrasound probe often elicits significant tenderness. Laboratory findings may further support the diagnosis, with elevated inflammatory markers and negative tumor markers.

Our case reflects current literature trends showing an increasing incidence of early-onset CRC, often presenting as symptomatic, left-sided, and advanced disease. (14) Similar to other reported cases, timely multidisciplinary management—including chemotherapy during the second and third trimesters—was essential in balancing maternal treatment needs with fetal safety. In our patient’s case, chemotherapy administered during pregnancy, led to disease stabilization and a favorable neonatal outcome, aligning with evidence that such regimens can be used safely in pregnancy with appropriate monitoring. (17, 18).

Regarding timing of delivery, the patient’s compromised clinical condition at presentation did not allow us to confidently rule out the possibility of urgent preterm birth; therefore, antenatal corticosteroids for fetal lung maturation were administered. As her condition stabilized, we prioritized continuation of therapy and prolonged gestation, ultimately achieving a term delivery. This case reinforces that, whenever clinically feasible, term birth offers the best neonatal outcomes and should remain the primary goal.

Although the neonatal outcome was reassuring and the newborn did not require intensive care, we acknowledge that our follow-up is currently limited to the short term. Reports have described the presence of platinum–DNA adducts in children exposed *in utero* to DNA-damaging agents such as cisplatin, carboplatin, or oxaliplatin, raising the possibility of long-term effects that remain insufficiently understood. (19, 20) Continued pediatric surveillance will therefore be essential to assess potential late consequences of fetal chemotherapy exposure.

Overall, this case further supports the role of a tailored, team-based approach in managing cancer during pregnancy. It highlights the feasibility of administering chemotherapy after the first trimester, achieving disease control while allowing for fetal maturation and delivery at term.

## Conclusions

This case highlights the complexity of diagnosing and managing colorectal cancer during pregnancy, particularly in advanced stages. Despite the challenges, timely multidisciplinary coordination allowed for effective maternal treatment with chemotherapy during the second and third trimesters, leading to significant tumor response and the delivery of a healthy newborn at term. This experience reinforces the feasibility and safety of chemotherapy in selected pregnant patients and underscores the importance of individualized care to optimize both maternal and fetal outcomes.

## Data Availability

The original contributions presented in the study are included in the article/supplementary material. Further inquiries can be directed to the corresponding author.
